# Human Milk Oligosaccharides: Health Benefits, Potential Applications in Infant Formulas, and Pharmacology

**DOI:** 10.3390/nu12010266

**Published:** 2020-01-20

**Authors:** Michał Wiciński, Ewelina Sawicka, Jakub Gębalski, Karol Kubiak, Bartosz Malinowski

**Affiliations:** 1 Department of Pharmacology and Therapeutics, Faculty of Medicine, Collegium Medicum in Bydgoszcz, Nicolaus Copernicus University, M. Curie 9, 85-090 Bydgoszcz, Poland; wicinski4@wp.pl (M.W.); jakubgebalski@gmail.com (J.G.); bartosz.malin@gmail.com (B.M.); 2 Department of Obsterics and Gynecology, St. Franziskus Hospital, 48145 Münster, Germany; dr.karolkubiak@gmail.com

**Keywords:** human milk oligosaccharides, bifidobacteria, prebitics, infections, nutrition

## Abstract

The first months of life are a special time for the health development and protection of infants. Breastfeeding is the natural and best way of feeding an infant, and positively influences their development and health. Breast milk provides the ideal balance of nutrients for the infant and contains countless bioactive ingredients such as immunoglobulins, hormones, oligosaccharides and others. Human milk oligosaccharides (HMOs) are a very important and interesting constituent of human milk, and are the third most abundant solid component after lactose and lipids. They are a structurally and biologically diverse group of complex indigestible sugars. This article will discuss the mechanisms of action of HMOs in infants, such as their anti-adhesive properties, properties modulating the immune system, and impact on bacterial flora development. Many health benefits result from consuming HMOs. They also may decrease the risk of infection by their interactions with viruses, bacteria or protozoa. The commercial use of HMOs in infant formula, future directions, and research on the use of HMOs as a therapy will be discussed.

## 1. Introduction

**Figure 1:**
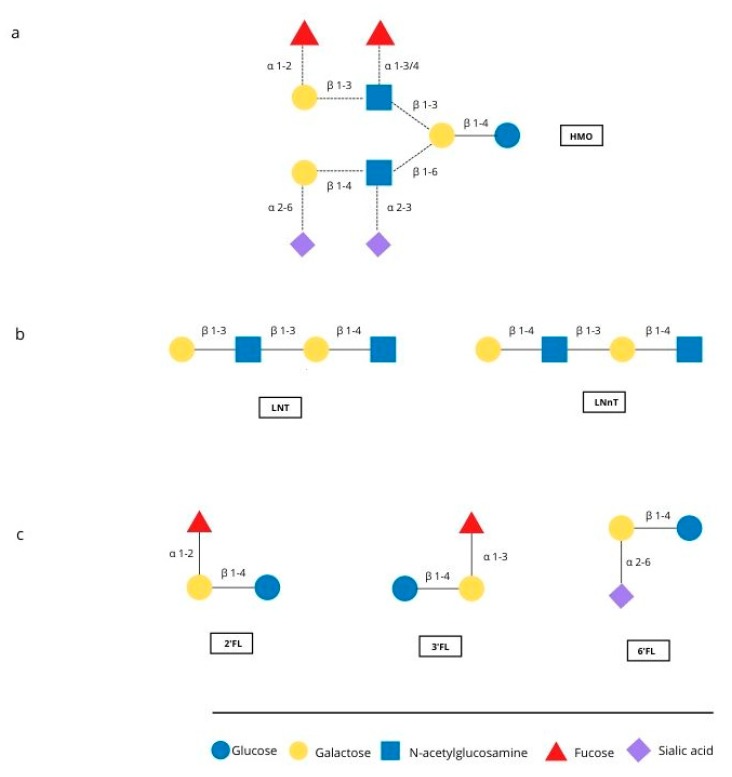
Representative structure of HMO and the major oligosaccharides found in breast milk. (**a**) Possible linkages of HMO building blocks, (**b**) type 1 (LNT) and type 2 chains (LNnT) (**c**) structures of 2′FL, 3′FL and 6′FL.

The first months of an infant’s life are a special time, with rapid development occurring. Breastfeeding is the natural and best way of feeding an infant, and positively influences the development and health of the infant. Human milk is the perfect food for infants, called “living tissue” by many [1,2] because it not only maintains an ideal balance of nutrients but also contains countless bioactive ingredients such as immunoglobulins, hormones, oligosaccharides and other components [3,4]. One important component is human milk oligosaccharides (HMOs), which are multifunctional glycans, naturally present in human milk. They are particularly interesting because of their quantity and structural diversity. About 15 structure of HMO have been identified in human milk [5]. Figure 1 shows represantive structures and major oligosaccharides. HMOs are made of five basic monosaccharides: glucose (Glc), galactose (Gal), N-ethylglucosamine (GlcNAc), fucose (Fuc) and sialic acid (SA). Almost all HMOs contain lactose (Gal-B1, 4-Glc) at the reducing end, which can be extended with lacto-N-biose I (Gal-b1, 3GlcNAc) or lactosamine (Gal-b1, 4-GlcNAc). Branching can be linear or branched through bonds b1-3 or b1-6. The sequence can be further modified by the addition of Fuc and/or SA monosaccharides through alpha 1-2,3,4 and alpha 2-3,6 bonds due to the action of fucosyltransferases and sialyltransferases [6].

Human milk contains three major HMO types: neutral, neutral N-containing and acid. Examples of these HMOs and the frequency of occurrence in milk are described in the Table 1. All HMOs are based on a lactose molecule (a disaccharide composed of a galactose molecule connected by a β1, 4-glycosidic bond to a glucose molecule) so it is likely that HMO biosynthesis is an extension of lactose biosynthesis. Lactose synthesis takes place in the Golgi apparatus of epithelial cells and is catalyzed by lactose synthase (LS). This has been well described, but oligosaccharide synthesis needs further exploration. HMOs are resistant to low pH in the stomach and pancreatic brush enzymes [7]. Studies on HMO metabolism have shown that 1% is absorbed into the circulation and the rest are metabolised by gut microbes or excreted in faeces and urine [8]. Quantitative measurement of HMOs is difficult due to their biological variability and lack of standards. We currently do not have particularly sensitive and specific methods. Studies have shown that the amount of HMO in milk can be different between women and also during different stages of lactation [7,9,10].

Colostrum is the first food of every newborn mammal, including humans. It is a thick, yellow liquid secreted by the mammary gland. It begins to form in the mammary glands during pregnancy. The highest concentration of HMO occurs in colostrum and amounts to 20–23 g/L, and then falls in mature milk to 12–14 g/L [11]. The milk of mothers who have given birth to premature babies has higher HMO concentrations than the milk of mothers who gave birth at term [12].

Mothers can synthesize various HMOs based on their genetic background. The most important example is related to the Lewis system and secretory status. The differences are in the presence or absence of fucose in the oligosaccharides.

Two fucosyltransferases—FUT2 (encoded by the secretory gene based on the 19q13.3 gene) and FUT3 (encoded by the so-called Lewis gene based on the 19p13.3 gene)—play a key role in HMO fucosylation. Both of these genes are expressed in the glandular epithelium [14]. Seventy-nine percent of mothers have the active gene for fucosyltransferase (FUT2). Table 2 shows 4 groups of mothers with different fucosylated HMO profiles.Twenty-one percent of mothers do not have the functional FUT2 enzyme and produce milk without the α1,2-fucosylated oligosaccharides 2′FL and LNFP I [5,8,15].

Lack of FUT3 enzyme can have negative consequences. Infants of women who do not have the FUT3 enzyme show delayed colonization of *Bifidobacteria*, and show more differences in the metabolic activity of their microflora, especially *Streptococcus*.

We can also distinguish four phenotypes based on the Lewis system, which are mentioned in the Table 3.

There is a relationship between the presence of specific phenotypes in the Lewis system and the tendency for complications after exposure to pathogens. For example, Leb antigens are receptors for *Helicobacter pylori*. This is especially important for people with blood type 0 because they have a higher presence of Leb antigens [16]. This is important because it has been shown that people with the Le (a^+^b^−^) and Le (a^−^b^−^) phenotype are particularly at risk of infection with cholerae-type O1. People with the Le (a^−^b^+^) phenotype have a much lower probability of infection, and if it does occur, hospitalization time is reduced and they have better outcomes. In contrast, people with the Le (a^−^b^−^) phenotype have a reduced number of IgA antibodies to this virus (also compared to Le (a^+^b^−^)). This causes diarrhea, which resolves slowly [17].

## 2. Infant Dietary Products

HMOs are absent in infant formulas, and so babies on these formulas do not receive the health benefits of consuming HMOs. Mixtures of galactooligosaccharides (GOSs) and fructooligosaccharides (FOSs) or inulin, known as bifidogenic, were therefore developed. These compounds may mimic the prebiotic from mother’s milk and enter the composition of the intestinal microflora, making them similar to breast milk [18,19]. HMOs are complex glycans composed of five different monosaccharides, while FOSs and GOSs are much simpler structures. FOSs are linear polymers of fructose and GOSs contain lactose at the reducing end, which is usually extended to six Gal residues that may contain different branches ([Gal (β1–3/4/6)] 1–6 Gal (β1–4) Glc). FOSs can be variable, depending on the method used. The first method uses the inverse fructanase and sucrase reaction or enzyme hydrolysis of inulin [19], which gives short chain FOSs. They have no reducing end and contain one Glc residue, and two or more fructose residues. The second method produces long chain FOSs. It consists of the hydrolysis of inulin, causes free anomeric carbons and contains one fructose. GOSs are mainly produced by enzymatic treatment of lactoseβ-galactosidase from fungi, yeasts or bacteria [20]. A mixture of oligosacharides with different oligomer chain lengths is obtained from this process. Studies in mice also found that mixtures supplemented with GOSs and FOSs can modulate the immune system [21], reducing the incidence of infections and atopic dermatitis. GOSs and FOSs are different structurally from HMOs found in human milk. Adding GOS and FOS or inulin is a reasonable and inexpensive way to add oligosacharides to formula, improving the quality of baby formulas. Much research is still needed to clarify the specific effects of GOSs and FOSs. Many mechanisms of natural HMO action still require explanation.

Two HMOs, 2′-fucosyllactose (2′FL) and lacto-N-neotetraose (LNnT), have recently been added to infant formula. Indeed, a study showed that the composition of the microflora of infants fed a 2′FL (1 g/L) and LNnT (0.5 g/L) supplemented formula was different from that of infants fed without milk, where the former group had flora more similar to those breastfed at 3 months of age; *Bifidobacterium* was more abundant, while *Escherichia* and *Peptostreptococcaceae* were less abundant. Fecal concentrations of several important metabolites such as propionate, butyrate and lactate in infants fed the HMO supplement, were more similar to those in breastfed infants [20,22].

## 3. HMO Mechanism of Action in Building Resistance

### 3.1. Prevention of Pathogen Adhesion

Evidence for an anti-adhesive effect of specific HMO comes from in vitro and ex vivo studies. HMOs serve as soluble ligand analogs and block pathogen adhesion. Many viruses or bacteria must attach to epithelial cell surfaces to proliferate and cause disease. Usually, the first attachment is to epithelial sugars on the cell surface (glycans), also called the glycocalyx. HMOs resemble some glycan structures and serve as soluble luring receptors that then block the pathogen binding to epithelial cells. Unbound pathogens are unable to attach to the cell surface and are excreted without causing disease.

HMOs seem to have glycomic modifying effects by changing glycan expression on the surface where many pathogens and commensal bacteria attach. Caco-2 cells have been shown to change their surface glycan profile after exposure to the 3′SL component of HMOs. Therefore, it is possible that this particular HMO modifies the glycan content on the surface of epithelial cells and receptor sites for some pathogens [7,8].

Pathogen adhesion is often initiated by lectin–glycan interactions, which have been described for many viruses such as noroviruses or rotaviruses. Similarly, *Escherichia coli* with type 1 fimbriae (fimbria has the property of binding to the host protein) bind to mannose-containing glycans, whereas *E. Coli* and *S. fimbriae*, as well as *Helicobacter pylori*, bind to sialylated glycans. Studies in a mouse model have revealed that 2′FL attenuates *C. jejuni* invasion by 80% and inhibits the release of mucosal pro-inflammatory signals. The beneficial effect of 2′FL is thought to include a reduction in the number of diarrhea episodes associated with *C. jejuni*. LNnT has been shown to reduce the number of *Streptococcus pneumonia* cells in the lungs of an animal model [7]. There is a unique antibacterial role for HMOs against the leading neonatal pathogen *Streptococcus* B. HMOs may act as a substrate to modify growth of these bacteria [23]. The anti-adhesive properties of HMOs also apply to some parasitic protozoa, e.g., *Entamoeba histolytica*, which cause amoebic dysentery or amoebic liver abscess. This protozoa destroys the epithelium of the large intestine. In addition to the intestinal wall, it can also occur in the liver, lungs or spleen. Around 50 million people worldwide are infected with *E. histolytica*, resulting in around 100,000 deaths annually. This is the third leading cause of death from a parasitic disease [24]. *Entamoeba histolytica* infection requires attachment to the host’s colon mucosa. Parasites that cannot attach are excreted in faeces and do not cause disease. HMOs are only minimally digested and absorbed in the small intestine and therefore reach the colon at the same site as *E. histolytica* infection. Some HMOs significantly reduce the binding and cytotoxicity of *E. histolytica* during in vivo assays [25]. This may explain why breastfed infants are less likely to be infected with *E. histolytica* than formula-fed infants.

### 3.2. Effects of HMOs on Microbiota Composition

The development of the intestinal microflora of infants is a sequential process. The beginning of this process is considered to be fetal life and the end is at about 3 years old. At this time, the digestive tract is colonized by bacteria, mainly from the Enterobacteriacae family, especially from the *Escherischa coli*, *Klebsiella*, *Enterobacter, Bacteroides*, and *Clostridia* groups. In infants who are breastfed, bifidobacteria predominate. There are fewer bacteria of the genera *Clostridium* and *Enterococcus*, while the least are *Klebsiella* and *Enterobacter*. In those infants that are fed artificially, the microflora resembles the digestive tract of adults and hence its composition is more complex than those infants who are breastfed. The dominance of bifidobacteria is the result of the presence of a bifidogenic agent in the food consumed. Oligosaccharides are such a factor. In the large intestine, they are fermented by bifidobacteria. The main product of this fermentation is acetic acid, which reduces the pH in the intestine. It is bacteriostatic, inhibiting the growth of pathogenic bacteria. In addition to acetic acid, fermentation products include butyric and propionic acid. They also have an important function. Butyric acid is an important source of energy for colonocytes. HMOs can indirectly increase short chain fatty acid (SCFA) production, and these elevated levels are mediated by bifidobacterial species. SCFAs are an important source of energy for enterocytes and are key molecules for maintaining intestinal health. Acetate, butyrate and propionate are dominant. Lactate and succinate, which are intermediate metabolites in SCFA production, are also present but are less studied. In adults, most SCFAs are rapidly absorbed or used by colonocytes as an energy source [26]. There is increasing evidence that SCFAs have a wider systemic effect because they act as signaling molecules and are involved in the regulation of gene expression [27]. It is believed that SCFAs are associated with appetite suppression by activating free fatty acid receptors in the intestine and increasing circulating intestinal anorectic hormones [28]. SCFAs have also been shown to play an important role in the activation and differentiation of immune cells and are associated with inflammatory and allergic diseases [24,25,28,29].

The preferred composition of the intestinal microflora is one in which bifidobacteria predominate. This composition can be seen in breastfed infants. Attempts are being made to increase the amount in the large intestine by using probiotics. Prebiotics are defined as “a selectively fermented ingredient that allows specific changes in both composition and/or activity in the gastrointestinal microflora, which provides benefits to the welfare and health of the host” [30,31]. Prebiotics must be resistant to stomach acidity, hydrolysis by host enzymes and gastrointestinal absorption. HMOs meet all three criteria. The vast majority of HMOs reach the distal small intestine and colon intact and in high concentrations [7]. Currently, prebiotic characteristics have been confirmed for indigestible oligosaccharides—fructose derivatives (FOS) and galactose (GOS). Their current application and properties will be discussed later in the article.

### 3.3. Antiviral Activity

Viral infections pose a serious threat to human health. Vaccines exist for influenza or rotavirus, but other viruses are still a problem. The main obstacle to developing effective vaccination is a virus evolution that generates new antigen variants. There are very few anti-viral drugs, and therefore, most viral infections cannot be cured. Long-term treatment of viral infections can cause undesirable effects or induce resistance strains [32,33,34].

HMOs have a high potential to provide protection against several viral pathogens [35,36,37]. They can act as an antiviral through a number of mechanisms, which were described earlier. They promote the maturation of the immune system and create a more balanced Th1/Th2 cytokine response. They may stimulate the immune response and maturation of epithelial cells to protect the host against virus infection. They affect the diversity and concentration of microbiomes and stimulate the growth of commensal bacteria. Another antiviral activity of HMOs is based on the fact that organism’s structure resembles the structure of various cell surface carbohydrates. Because of this, HMOs catch viruses that do not stick to cells. This mechanism is based on the structural similarity of HMOs to the sugar chains of glycoconjugates present on the surface of epithelial cells. HMOs mimic the surface glycans of epithelial cells. Soluble fucosylated and sialylated human milk oligosaccharides can be recognized and bound by lectin receptors of fucose and/or sialyl-dependent bacteria, and/or by lectin receptors present on the surface of host epithelial cells. In both cases, fucosylated and/or ovalized HMOs catch viruses and participate in blocking lectin receptors. Virus lectin receptors blocked by HMOs cannot participate in the recognition of glycotopes present on the surface of host cells, which prevents their adhesion and colonization.

A mixture of sialylated HMOs reduces selectin-mediated neutrophil rolling and adhesion in vitro, as well as PNC formation and neutrophil activation ex vivo [38,39].

The discovery of HMO protection against rotavirus and norovirus infections can provide an alternative to current therapies. HMO action is directed towards more conserved capsid proteins. Hemaglutinin could potentially be used to treat influenza as an HMO-based medicine. Mono-sialylated HMO (3′SL and 6′SL) has been shown to cause reduced influenza infection in cell culture assays. Nevertheless, more data is still needed for the acquired knowledge to be used for therapeutic purposes. Recent reports that HMO may reduce HIV-1 mother-to-child transmission in breastfed infants are very interesting [40,41].

### 3.4. Norovirus

Noroviruses belong to the calicivirus family. This group includes viruses formerly defined as Norwalk, Norwalk-like, Sapporo, and Sapporo-like viruses. Now, at least four of their genotypes, GI, GII, GIII and G IV, are known. Caliciviruses also belong to the food-borne hepatitis E virus. Noroviruses are recognized as one of the most important causes of infection of unknown viral etiology, especially in adults. Debilitated and elderly people can become infected with these viruses, leading to deaths caused directly by dehydration [42].

Over 10 years ago, scientists identified putative human norovirus cell receptors called histone blood group antigens (HBGAs). HBGAs are complex terminal carbohydrates present in cells and secreted into body fluids [43]. The interplay between HBGAs, HMOs, and norovirus capsid proteins has been extensively studied in recent years. It has been shown that the addition of HMOs to foods can help fight noraviruses. HMOs work by preventing its binding to epithelial surfaces. HMOs mimic the structure of blood-active mucin-type O-glycans. They are an ideal source of potential competitors for viral glycan receptors; however, knowledge of HMOs is still limited. 2-fucosyllactose (2′FL) trisaccharide is one of the smaller but more common HMOs. It drew the attention of scientists because it is able to quite effectively block norovirus binding [44,45]. 2′FL is registered as a safe food additive. It is not known, however, whether other more complex glycans in the high mass HMO fraction may contain components with greater competitive activity in binding norovirus, and whether these glycans can give hints as to the fine structural requirements for binding norovirus to the host epithelium [46].

#### 3.4.1. Rotavirus

Rotaviruses belongs to the Reoviridae family and contain a double RNA chain, enclosed in a three-layered, non-enveloped, 20-walled capsid. Of the seven main rotavirus groups (A to G), human disease is primarily caused by group A rotaviruses, and much less frequently by groups B and C. Two surface proteins VP7 (G protein) and VP4 (P protein) determine the specificity of the serotype basis for rotavirus classification (types G and P) [47]. Rotavirus infection is the main cause of gastroenteritis and diarrhea in infants and young children and accounts for 5% of all deaths in children <5 years old. Breastfed infants have a lower incidence of acute rotavirus-induced gastroenteritis than infants fed formula-modified milk [48,49].

Like human noroviruses, a person’s blood group status has now been correlated with susceptibility to some rotavirus genotypes. Some individuals are generally more susceptible to rotavirus infection than others [44,50,51]. For example, the high incidence of Lewis positive phenotype in the population may explain the lower occurrence of rotavirus P [16]. Rotaviruses interact with HMOs in a similarly selective manner. Direct evidence for the association of rotaviruses with HMOs has been shown in animal studies. Newborn pigs were fed with HMOs (2′FL, lacto-N-neo tetraose, 6′-sialyllactose, 3′-sialyllactose and free sialic acid) or a mixture containing short-chain galacto-oligosaccharides (GOSs) and long-chain fructo-oligosaccharides (FOSs). They were then inoculated with a pig rotavirus strain on day 10. The duration of rotavirus-induced diarrhea was also shortened in piglets fed a mixture of GOS and FOS. Pigs that received HMO treatment had almost twice as many NK cells and five times as many basophils as mixed-fed pigs [45].

Studies have also been performed on a monkey epithelial cell line (MA-104) that was mixed with HMOs consisting of 3′SL, 6′SL, 2′FL and galactooligosaccharides (GOSs). This study showed that both sialylated and fucosylated oligosaccharides can reduce the infectivity of human rotaviruses. It is thought that many human rotavirus strains, including G1P [9], use internal sialic acid residues on cell surfaces as receptors. However, HBGA is increasingly being considered as an important human binding agent for rotaviruses. Specifically, fucosylated glycans, including H-type 1 and Lewis b HBGA, are currently recognized as potential binding partners for both G1P [9] and G2P [4,52,53]. This study showed that all oligosaccharides tested in this study can reduce the infectivity of human rotavirus strains G1P [9] and G2P [54].

#### 3.4.2. Respiratory Viruses

Influenza viruses belong to the Orthomyxoviridae family. Types A, B and C are distinguished among them. Of all its representatives, only type A viruses have the potential to infect a wide spectrum of hosts: both animals and humans. Different strains are characterized in this group due to the different antigenic properties of the two surface, spiny glycoproteins: hemagglutinin (HA) and neuraminidase (NA). The genes encoding HA and NA are very variable, resulting in only 30% of the amino acid sequence being conserved within all their subtypes. Currently, 16 different HA and 9 NA subtypes have been identified [55].

When consumed, HMO bathes the laryngopharyngeal region. It was postulated that it may reduce pathogen adhesion at the entrance to epithelial cells of the respiratory mucosa [15]. This also applies to locally residing or transient-immune cells (lymphocytes, dendritic cells, monocytes, macrophages, NK cells and M cells) in the palate and lingual tonsils. HMO, with the participation of neutrophils, has been shown to suppress platelets and the inflammatory response. It is absorbed into the circulation with potential access through the tonsils or intestinal mucosa [56,57,58,59].

HMO interaction studies were performed with respiratory epithelial cells or peripheral blood mononuclear cells. The effect of HMOs on the course of viral infection and cytokine expression was observed. 2′FL has been shown to reduce RSV viral load, while LNnT and 6′SL reduce the influenza viral load. It has been concluded that HMO at or below the level found in breast milk increases innate immunity to respiratory viruses in vitro and can affect innate immunity. As a result of blocking hemagglutination of influenza viruses, immobilized 3′SL and 6′SL attachments prevent infectivity of influenza viruses [60,61]. A number of external HMO connections have been identified that bind to influenza virus [60,61]. A number of additional sialic acids containing HMO that bind to influenza virus have also been identified. One interesting study in vivo showed that 2′FL enhanced responses to vaccination in mice. Administration of 2′FL in mice improved the humoral and cellular immune response to vaccination. It is thought that this is a direct 2′FL effect on the differentiation of immune cells [62].

#### 3.4.3. Human Immunodeficiency Virus

HIV is a RNA virus in the Lentivirus genus of the Retroviridae family. Any HIV infection leads to the acquired immunodeficiency syndrome (AIDS). The virus targets CD4+ Th lymphocytes, macrophages, monocytes and dendritic cells [54]. Infection occurs through sexual or perinatal transmission, or as a consequence of exposure to secretions or tissues containing the virus. The infectious material is blood, semen, pre-ejaculate, vaginal secretion, rectal secretion, milk, and unfixed tissues. HIV-1 virus can be transmitted from the mother during breastfeeding. However, only about 10–15% of infants fall ill from a mother infected with HIV through breastfeeding when breastfeeding exclusively [63]. The HIV virus binds to the dendritic cell-specific intercellular adhesion molecule (DC-SIGN) on human dendritic cells that carries the virus across the mucosal barrier. The most likely mechanism of HMO action on disruption of HIV infection is that HMOs interfere with the binding of HIV to DC-SIGN. DC-SIGN also has a strong affinity for Lewis blood group antigens, which form part of the HMOs in the milk of some women [40].

### 3.5. Immunity System Development

The newborn’s immune system differs from the adult’s immune system because it is functionally naive and has a different quantitative and qualitative composition. The differences are important for newborns under 1 month of age because breastfeeding reduces the incidence and alleviates the course of infectious diseases, which affects the survival of the newborn. Breastfeeding for the first six months of an infant’s life has been shown to affect the maturity of the immune system. There is evidence that exclusive breastfeeding reduces the incidence of many diseases such as asthma, allergies, inflammatory bowel disease, type 1 diabetes, celiac disease and leukemia.

HMOs are a very important component of breast milk as they can affect the infant’s immune system through various mechanisms. The composition of the infant microbiome and the HMO-mediated epithelial cell responses may indirectly affect the infant’s immune system. However, many in vitro studies have suggested that HMOs can also directly modulate the immune response. HMOs may act locally on lymphoid tissue cells associated with the mucosa, and at the systemic level, as 1% of the HMOs is absorbed and reaches the systemic circulation. HMOs are detected in the plasma of infants fed human milk in concentrations of 1–133 mg/L, therefore it is believed that HMOs in the diet can directly affect the immune system [64,65].

Many immunoreceptors recognize the oligosaccharide structures of their glycoprotein ligands. HMOs are structurally similar to selectin ligands, so it is believed that they may bind directly to immune cells [66,67]. Such binding can cause signaling that leads to changes in the populations and functions of immune cells. During inflammatory processes, E and P selectin, present on the surface of endothelial cells, recognize and participate in interactions with sialyl-Lewis X glycotopes (sLeX). sLeX is a member of the glycoconjugates, on the surface of leukocytes, which are one of the elements involved in the process of leukocyte extravasation and mucosal infiltration [11]. HMO enzymatic modifications such as fucosylation and sialylation allow binding to selectins [68].

Umbilical cord blood T cells exposed to sialylated HMO cause an increase in the number of CD3+/CD4+ and CD3+/CD8+ cells producing γ interferon, and CD3+/CD8+ cells producing interleukin-13 (IL-13). This is important because sialylated HMO is thought to affect lymphocyte maturation and promote the shift of T-cell responses towards more balanced Th1/Th2 cytokine production and low immunity. Some sialylated HMOs have been shown to contribute to the prevention of allergies, such that sialylated HMOs reduced IL-4 production in the lymphocyte subgroup from adult patients with a peanut allergy [56,66,67,69].

The effect of HMOs from colostrum on fetal human intestinal mucosa cells has been investigated. A cell model with intestinal epithelial cells (T84/HCT8/FHs74) and HeLa cells was used for this purpose. The result of the research was characterization of the networks controlling the communication of immune cells, differentiation of the intestinal mucosa immune system and homeostasis. It has also been found that the use of HMOs reduced the levels of cytokine proteins such as IL-8, IL-6, monocyte chemoattractant protein 1/2 and IL-1β. In contrast, the level of cytokines involved in tissue repair and homeostasis increased.

## 4. NEC

Necrotising enterocolitis (NEC) leads to severe and often fatal destruction of the infant’s intestine. It affects 5–10% of premature babies with low birth weight (less than 1500 g). More than 25% of infants with NEC die. Infants who survive, often have long-term neurological complications. Breastfed infants have a 6 to 10 times lower risk of developing NEC than formula-fed infants [70,71]. The etiology and pathogenesis of NEC remain poorly understood. Treatment is limited and based on cessation of enteral nutrition, antibiotic therapy, and, in severe cases, surgical removal of necrotic intestine, which can be accompanied by long-term complications. Although infant milk formulas have improved in the last 10–15 years, the difference in NEC risk between formula-fed and breast-fed infants remain unchanged. It has been concluded that HMOs contribute to the protective effects of human milk against NEC. Studies conducted on a rat model have shown that HMOs do protect against NEC. If these results translate to NEC in humans, disialyllacto-N-tetraose (DSLNT) could be used to prevent or treat NEC in formula-fed infants, and its concentration in the mother’s milk could serve as a biomarker to identify breastfed infants at risk of developing this disorder. It may also be useful for developing innovative therapies against NEC. Additional research and DSLNT effects in NEC extension should be performed. There are currently no data in infants confirming the effect seen in animal models [72,73].

## 5. New Possibilities of HMO Applications

Considering the action and therapeutic potential of HMOs, other applications have been sought. A study was carried out to check whether a GOS/FOS mix had effects on bone health. It was found that it could help maintain bone health by reducing bone resorption, and increasing bone mineralization, density and structure with a simultaneous increase in absorption of Ca, Pi and Mg. The results of these studies are very promising, although studies are still needed that would confirm the effectiveness of the GOS/FOS mix. If the results are confirmed, the GOS/FOS mixture may be used in the future in combination with traditional pharmacological treatments [74].

HMOs are found in the plasma and urine of breastfed infants. Although the “normal” concentration has not yet been determined, HMOs have been found in the urine of pregnant women at the end of the first trimester. Test results confirm the postulate that HMOs result in fewer cases of GBS infections in breastfed infants during pregnancy due to the anti-bacterial properties of HMOs. Studies have also shown synergy of HMOs with some antibiotics, so they may be useful in the treatment of GBS infections. Future research can help identify the relationships of individual HMOs, such as LNT, with the risk of GBS infection. The development of new anti-infective strategies based on the natural standard of human milk seems likely [23].

Researchers studied the effects of oligosaccharides on constipation in mice. The aim of this study was to assess the effects of three different types of oligosaccharide—FOS, GOS and IMO on loperamide-induced constipation. Oligosaccharides were administered intragastrically to healthy mice once a day for 17 days. The indicators of constipation, changes in intestinal microflora and metabolic activities were analyzed, and oligosaccharides were shown to treat constipation. They increase the water content in the feces, reduce the time of intestinal transit, modulate the composition of the intestinal microflora and increase the concentration of short chain fatty acids in the feces of mice with constipation. After oligosaccharide treatment, Lactobacillus and Bifidobacterium dominated in intestinal microbiota and decreased levels of Odoribacter, Alistipes and *Bacteroides* were found [75].

HMO supplementation studies were conducted in 100 healthy adults consuming 2′-O-fucosyllactose (2′FL) and/or lacto-N-neotetraose (LNnT) at various daily doses and mixtures, or placebo, for 2 weeks. It was shown that 2′FL and LNnT supplementation in daily doses up to 20 g are safe and well tolerated. Analysis showed supplementation modified the microflora of adult intestines. The treatment changed the relative abundance of Actinobacteria and Bifidobacterium, as well as the reduction of Firmicut and *Proteobacteria*. This study is the first to show the safety, good tolerance and effects of HMOs on the intestinal microflora of adults. Thanks to the results of these studies, we can conclude that HMO diet supplementation may be a valuable opportunity to shape the human intestinal microflora, and especially to promote the growth of beneficial *Bifidobacteria*.

It has been suggested that HMOs may have therapeutic potential in allergic diseases. The effect of two HMOs, 2′FL and 6′SL, on anaphylactic effects was investigated by studying the symptoms caused by oral ovalbumin in an ovalbumin-sensitized mouse model of food allergy. The results of these studies suggest that 2′FL and 6′SL reduce symptoms of food allergy by induction of IL-10 (+) regulatory cells and indirect mast cell stabilization. Another study showed that the HMOs 2′FL and 6′SL modulate human epithelial cell responses. In particular, 6′SL may have additional antiallergic benefits because it inhibits the release of chemokines induced by antigen–antibody complexes and other inflammatory signals. This leads to inhibition of the influx of inflammatory cells into the intestines. These studies encourage further exploration of the therapeutic potential of HMOs in food allergies [76].

## 6. Conclusions

In conclusion, it can certainly be said that HMOs are a very important component of breast milk. They contribute to the development of the infant’s microflora and immune system. By acting via various mechanisms, they protect against many infections and alleviate their course. They have been shown to have anti-bacterial, anti-viral and anti-inflammatory effects. Exclusive breastfeeding up to 6 months is very important because it protects the health and life of infants. Many diseases, including diarrhea, respiratory and urinary tract infections, otitis media, bacteraemia and necrotizing enterocolitis are less common in breastfed children. Breastfeeding also has an impact on the course of other immune-related diseases such as celiac disease, asthma, allergy, type 1 diabetes, acute lymphoblastic leukemia and acute myeloid leukemia. It also reduces the incidence of these diseases.

Research on the possibility of giving infants ingredients that are functionally similar to HMOs, which are supposed to bring the same health benefits, is very important. Prebiotic oligosaccharides, which are a mixture of fructooligosaccharides (FOSs) and galactooligosaccharides (GOSs), are already used in modified milk. The potential of HMOs is larger than this and research in many areas is ongoing. However, faithful reproduction of breast milk composition is not possible at this stage of knowledge and a lot of research is still needed. Interdisciplinary teams consisting of chemists, pediatricians, nutritionists, microbiologists, glycobiologists and many others are needed.

## Figures and Tables

**Table 1:** Human milk contains three major HMO types [13].

Neutral (Fucosylated) HMO	35% to 50% of the Total HMO	e.g., 2′-Fucosyllactose (2′-FL) and Lactodifucopentaose
Neutral N-containing HMO	42% to 55% of the total HMO	e.g., lacto-N-tetraose
Acid (sialylated) HMO	12% to 14% of the total HMO	e.g., 2′-sialyllactose

Abbreviations: HMO, Human milk oligosaccharide.

**Table 2:** Groups of mothers with different fucosylated HMO profiles.

Lewis Positive Secretors (Se+Le+)	FUT2 Active	FUT3 Active
Lewis negative Secretors (Se+Le−)	FUT2 active	FUT3 inactive
Lewis positive Nonsecretors (Se−Le+)	FUT2 inactive	FUT3 active
Lewis negative Nonsecretors (Se−Le−)	FUT2 inactive	FUT3 inactive

**Table 3:** Phenotypes based on the Lewis system.

Le (a^+^b^+^)—Strong expression of the Lea antigen, but the Leb antigen is also synthesized with the Le and Se allel, strong expression of the Lea antigen, but the Leb antigen is also synthesized.
(a^−^b^+^)—Only Leb antigen is secreted, occurs in some people with the Le and Se alleles.
Le (a^+^b^−^)—Only Lea antigen is present, occurs in people with the Le all-dominant allele who are recessive homozygotes sese.
Le (a^−^b^−^)—Present in all lele homozygotes.
